# Comparison of Conventional Tube and Gel-Based Agglutination Tests for AB System Blood Typing in Cat

**DOI:** 10.3389/fvets.2020.00312

**Published:** 2020-06-23

**Authors:** Eva Spada, Roberta Perego, Luciana Baggiani, Daniela Proverbio

**Affiliations:** Veterinary Transfusion Research Laboratory (REVLab), Department of Veterinary Medicine (DIMEVET), University of Milan, Milan, Italy

**Keywords:** cat, blood group, blood-typing method, agglutination, tube, gel, comparison

## Abstract

Gel technology is widely used for blood typing in human medicine. It has a number of advantages over routine tube testing, including standardization, stability, smaller sample volume, ease of performance and analysis, and speed. The aim of this study was to evaluate feline blood typing using the gel column technique. TUBE agglutination typing was performed in 143 feline blood samples from blood donors and recipients, healthy and sick patients, and whole-blood units anticoagulated with ethylenediamine tetraacetic acid or citrate phosphate dextrose adenine. Plasma from type B cats was used as anti-A reagent, *Triticum vulgaris* lectin as anti-B reagent, and the control was saline solution. Agglutination in backtyping of types B and AB samples with type A red blood cells (RBCs) was used to confirm whether the samples were type B (presence of alloantibodies) or type AB (absence of alloantibodies). Blood typing in a neutral gel column technique (GEL) using the same anti-A and anti-B reagents was performed on duplicate samples. Sensitivity, specificity, positive likelihood ratio, negative likelihood ratio, positive predictive value, negative predictive value, overall accuracy, and Cohen κ coefficient (κ) for GEL were calculated, with TUBE considered the gold standard technique. Of 143 samples typed with TUBE, 98 (68.5%) were type A, 25 (17.5%,) type B, and 20 (14.0%) type AB. Backtyping confirmed the categorization of all types B and AB samples. Of these samples, gel testing produced 115 (80.4%) concordant results; a mixed-field agglutination pattern (layers of RBCs at both the top and at the bottom of the gel in either the A or B gel column) was seen in 27 samples, and one type B sample was misidentified as type AB. If the mixed-field pattern was interpreted as a negative result, 141/143 (98.6%) samples showed concordant results with an overall accuracy of the GEL of 100.0% for type A, 98.9% for type B, and 99.1% for type AB. Strength of agreement was very good (κ = 0.97). When the same anti-A and anti-B reagents are used, GEL is a sensitive and specific method for blood typing feline samples. Until additional studies have been performed, mixed-field patterns obtained in GEL testing should be classified as negative results.

## Introduction

The feline AB blood group system, characterized by three phenotypes: type A, type B, and type AB, is the predominant blood group system in cats (1). In cats, naturally occurring alloantibody exists against the antigen they lack. Type A cats have low-titer anti-B hemagglutinins and hemolysins. Type B cats have high-titer anti-A hemagglutinins and hemolysins. Type AB cats do not possess alloantibodies against either A or B antigens (2). These naturally occurring alloantibodies are responsible for transfusion reactions and neonatal isoerythrolysis in cats with incompatible blood types.

A knowledge of breed-related blood type and regional blood type epidemiology is useful when looking at the prevalence of different feline blood types. For example, the rare AB blood type is more common in some breeds, such as Ragdolls, in some countries (3). In addition, data on blood type epidemiology are useful when creating a blood bank by identification of breeds that may be preferentially recruited as blood donors. Maine Coon cats are large and quiet and have a high prevalence of A blood type (4). These data can be obtained by blood typing a large number of cats using a sensitive and specific test that can identify all three blood groups of the feline AB blood system.

Blood typing can be performed in-house or at a commercial laboratory. Currently, the most widely used in-house commercial blood typing kits include card agglutination tests and immunochromatographic tests (5–7). Commercial laboratories and blood banks usually employ the TUBE agglutination method.

The TUBE method is considered the gold standard for blood typing (8, 9). It requires the use of antigen-specific antisera and is performed by trained medical technologists. Despite being the gold standard in feline blood typing, the TUBE method has been difficult to standardize. In this test, the patient's red blood cells (RBCs) are incubated with antibodies against a specific blood type in a tube and then centrifuged and resuspended to assess for hemolysis or agglutination. The advantages of this method are that it can be completed in any practice, and there is no need for specialized equipment. The disadvantages are that it requires antigen-specific antisera, there is no universal protocol for completion and/or interpretation, it is time consuming (at least 1 h to complete), it requires advanced training, it produces subjective results, and it does not produce a stable reaction.

To overcome some of these disadvantages, gel technology has been widely used for blood typing in human medicine for many decades. The gel agglutination test (GEL) detects RBC antigen–antibody reactions using a chamber filled with polyacrylamide gel. The gel acts as a trap: free unagglutinated RBCs form pellets in the bottom of the tube (negative reaction); agglutinated RBCs remain at the top of the tube or are trapped in the gel (positive reaction). GEL testing has a number of important advantages over routine TUBE testing, particularly when testing large numbers of samples. These include standardization, stability, smaller sample volume, ease of performance and analysis, and rapidity. However, it does require specialized equipment.

Previously, a GEL test for feline blood typing was also available. It consisted of a dextran–acrylamide gel matrix impregnated with an antibody against the blood type of interest, and it was shown in previous studies to be the most accurate bedside test (99.4% accuracy in cats) for feline AB blood typing (5, 8, 9); unfortunately, this test is no longer available. Neutral human GEL columns with the addition of specific anti-RBC blood type antigen such as blood type 3, 4, 5, 7, Dal, and Kai reagents have been used in the last decade and proved useful in canine blood typing (10–12).

This gel agglutination technology could also be used for feline AB blood system blood typing. Therefore, the aim of this study was to evaluate the neutral human gel column technique in AB blood system feline blood typing, comparing the results with the TUBE gold standard technique.

## Materials and Methods

### Blood Samples

Ethylenediamine tetraacetic acid (EDTA)–anticoagulated surplus blood samples used for blood typing cats for clinical reasons (blood donor, blood recipient, blood typing before mating, or as part of preoperative evaluation) at the Veterinary Transfusion Research Laboratory (REVLab) of the University of Milan were used in this study. The blood was stored at 4°C and typed within 7 days of collection.

During consultations and prior to blood donation or transfusion, owners provided written consent for blood collection, use of blood samples, and use of data for scientific purposes. Ethical review and approval were not required for the animal study because only surplus EDTA blood samples drawn for routine diagnostic work were used.

For each blood sample tested, the cat's age, breed, and sex were recorded. When available, the cat's health status or underlying disease was also recorded. Packed Cell Volume (PCV) and autoagglutination were recorded.

Duplicate samples were blood typed using the same anti-A and anti-B reagents in both the TUBE and neutral GEL column technique.

### Blood Typing by Tube Technique

TUBE was performed with slight modifications as previously described (2, 13).

To prepare an RBC suspension for the TUBE method, 1 mL of anticoagulated blood was centrifuged for 10 min [1,600 g at room temperature (~20°C)]. Plasma was collected into a separate tube and stored for possible backtyping or other analysis. The RBC pellet was resuspended in 5 mL of isotonic 0.9% saline NaCl solution. The suspension was then recentrifuged and resuspended three times and finally reconstituted to a 5% RBC suspension.

Polyclonal antibodies contained in type B cat plasma (obtained from a type B blood donor, collected with CPD anticoagulant at a ratio of 1:7, stored at −20°C) were used as primary reagents for the detection of type A red cell antigens. *Triticum vulgaris* lectin [8 μg/mL, made by mixing 2 mg of stock solution with 250 mL phosphate-buffered saline (PBS), stored, aliquoted in 10 ml tube at −20°C] (Sigma-Aldrich Corporation, MERK KGaA Darmstadt, Germany) was used for the detection of type B RBC antigens as this lectin binds to the NeuAc terminal of the type B ganglioside (13). A 0.9% saline solution was used as a negative control and to test for autoagglutination.

**Figure 1:**
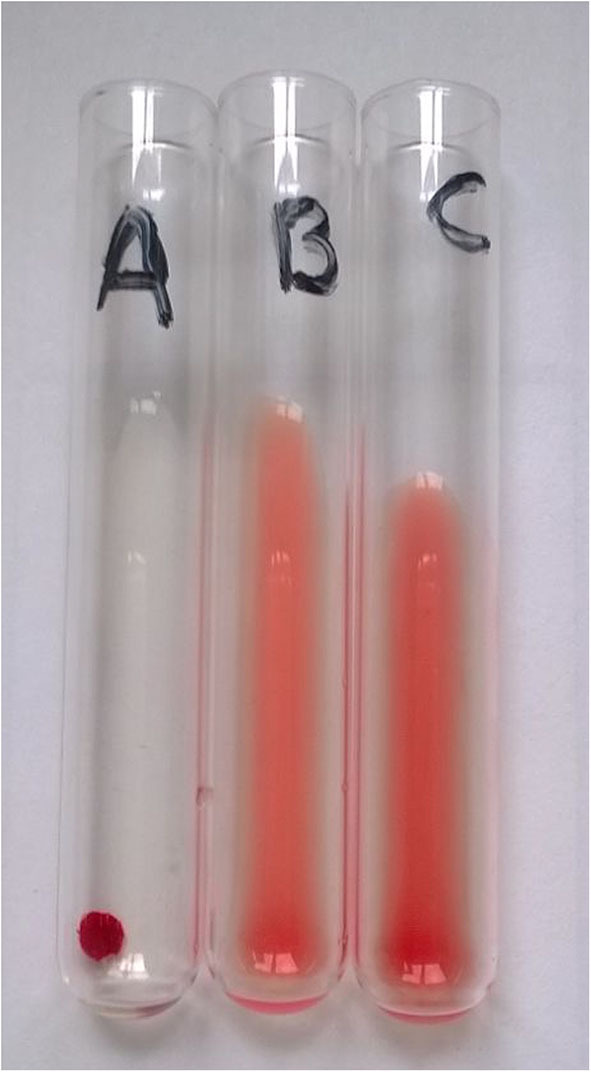
Example of type A blood sample typed using the tube agglutination technique (TUBE). Total clumping of red cells in “A” tube and absence of agglutination in “B” and “C” (control) tubes identify this sample as type A with maximum strength of agglutination (4+).

In three glass tubes, 50 μL of 5% RBC suspension was mixed with 100 μL of type B plasma (anti-A reagent), 100 μL of *T. vulgaris* lectin solution (anti-B reagent), or 100 μL of saline solution (control reagent), respectively. These mixtures were incubated at room temperature for 15 min before centrifugation for 15 s at 1,000 g. Tubes were then gently agitated, and agglutination was scored from 0 (no agglutination, negative reaction) to 4+ (single pellet-like agglutination, maximum grade of positive reaction, Figure 1).

### Blood Typing by Gel Method

Duplicate samples were blood typed with a neutral gel column technique using the same anti-A and anti-B reagents as in the TUBE technique. The GEL test consists of a card with six microtubes for determining the blood types of two samples. Each microtube contains a neutral gel matrix (ID-Card NaCl enzyme test and cold agglutinins; DiaMed GmbH, Cressier FR, Switzerland).

Red blood cells were first separated from plasma by centrifugation at 1,600 g for 10 min, and a 0.8% RBC suspension prepared, by suspending 10 μL of the RBC pellet in 1 mL of low ionic strength solution (ID-Diluent 2; DiaMed) provided by the same manufacturer.

A 50 μL RBC suspension from each sample was loaded on the top of three gel columns (reaction chamber) and mixed with 25 μL of type B plasma, 25 μL of *T. vulgaris* lectin stock solution, and 25 μL of PBS in the A, B, and negative control (ctl) columns, respectively. Control columns that contained gel with PBS only were included to detect potential false-positive reactions due to autoagglutination.

**Figure 2:**
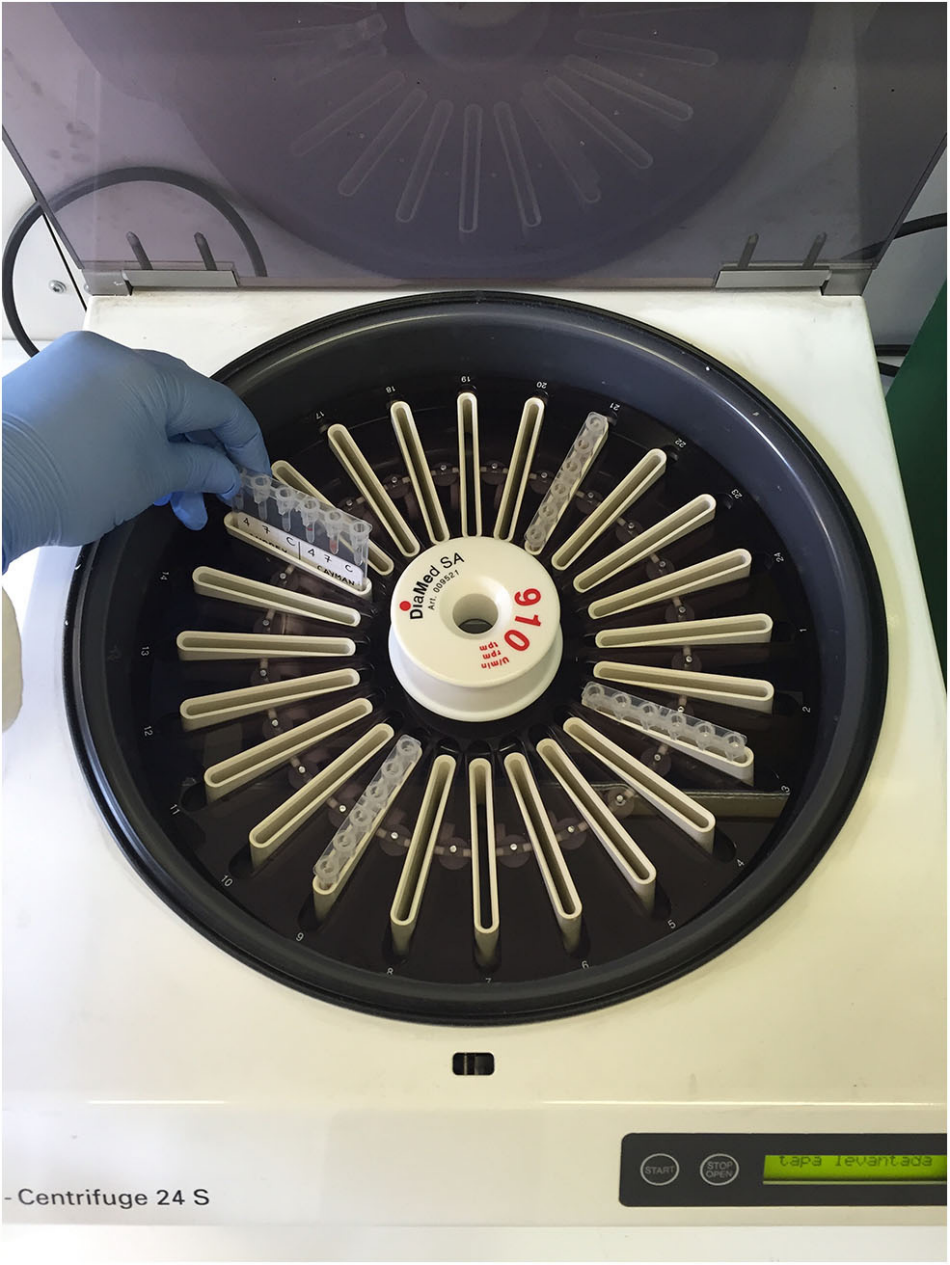
Special gel column card centrifuge in which the gel column cards were centrifuged at 80 g for 10 min.

**Figure 3:**
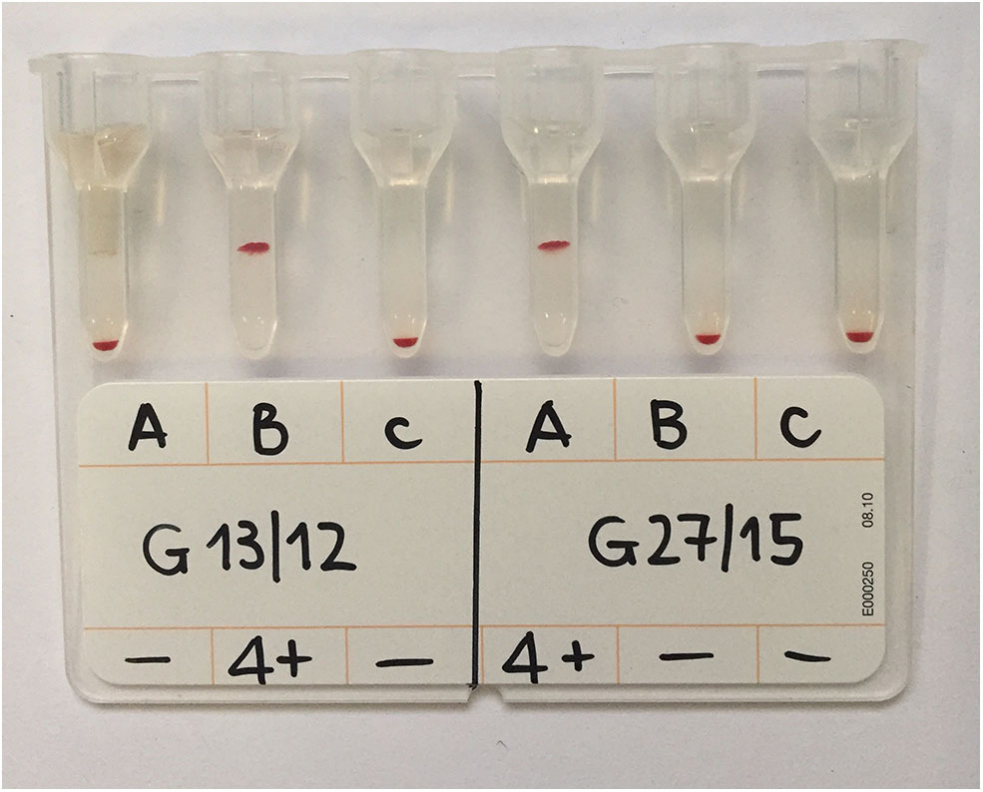
GEL column card with two analyzed samples. The sample labeled G13/12 was type B blood as the agglutination occurred only in the B column; sample G27/15 was type A as agglutination occurred only in the A column. Both reactions were graded 4+ as all the RBCs were agglutinated and formed a layer at the top of the gel column. Control columns (c) containing PBS only are included and show absence of autoagglutination.

**Figure 4:**
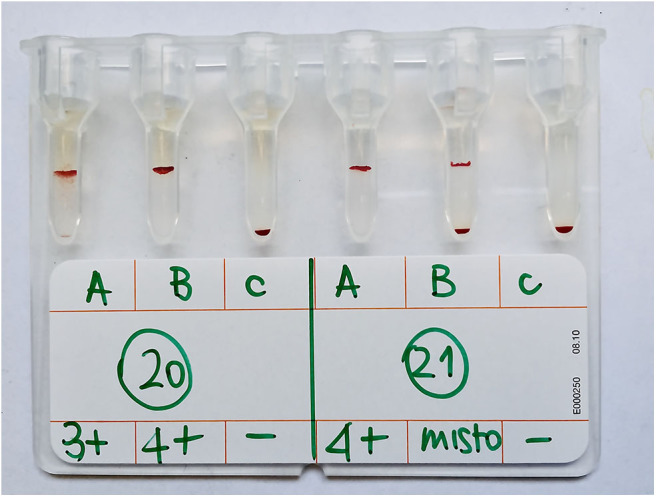
Example of samples with gel agglutination mixed-field pattern results. Sample *n*. 21 showed a mixed-field pattern (identified as “misto” in the Figure) in B column and was classified as A blood type based on comparison with agglutination on tube technique, the gold standard technique. Sample *n*. 20 showed agglutination in both A and B columns and was AB blood type, as confirmed by agglutination on tube technique. Control columns (c) containing only PBS are included and show negative test (absence of autoagglutination).

Following incubation at room temperature (~20°C) for 15 min, the gel columns were centrifuged in a special gel column card centrifuge (ID-Centrifuge 24 S; DiaMed; Figure 2) at 80 g for 10 min. The results were viewed and graded from negative to 4+ depending on the location of the majority of RBCs within the gel column, as follows: 4+: all RBCs were agglutinated and formed a red line on the surface of the gel; 3+: most RBCs were agglutinated and formed a red line on the surface of the gel; 2+: all RBC agglutinates were dispersed in the gel; 1+: few RBC agglutinates were dispersed in the gel, and most of the RBCs were at the bottom of the tube; 0 (negative): all RBCs were at the bottom of the tube (not agglutinated). If the A- or B-labeled column had a positive reaction, the cat had type A or type B blood, respectively; if both columns were positive, the cat had type AB blood (Figures 3, 4). Red blood cell retention of ≥1+ was considered a positive test result. The test was considered invalid if the ctl-labeled column was not consistently negative.

### Back Typing

Backtyping technique or antibody screening is a modified major crossmatch using RBCs of a known blood group, thus screening for recipient antibodies against that group.

To confirm types B and AB samples, all samples were tested for presence (type B) or absence (type AB) of anti-B isoagglutinins using a backtyping technique as previously described (3, 8, 9). Briefly, in a glass tube, 25 μL of the type A RBC suspension (5% in 0.9% NaCl) was added to 50 μL of type B or AB plasma. The solution was gently mixed, incubated for 15 min at room temperature (~20°C), and then centrifuged for 15 s at 1,000 g. Agglutination was evaluated by gently agitating the tube to resuspend the non-agglutinating RBCs in the cell button. Plasma from type B-positive cats showed a strong agglutination reaction with type A-positive RBCs, whereas plasma from AB-positive cats did not agglutinate with type A.

### Additional Analysis

To better understand the reasons for mixed-field pattern results in GEL, stored plasma samples (−20°C) of most blood samples showed this mixed-field pattern and available plasma from all other samples were retrospectively tested for feline leukemia virus infection (FeLV) and for feline immunodeficiency virus infection (FIV). Presence of antibodies to FIV target antigens p24 and gp40 and of FeLV p27 antigen was simultaneously checked on plasma using a commercial rapid enzyme-linked immunosorbent assay kit (SNAP® Combo Plus FeLV Ag/FIv Ab; IDEXX Laboratories Italia S.r.l., Milan, Italy).

### Statistical Analysis

As suggested by the National Committee for Clinical Laboratory Standards guidelines, a minimum of 40 samples from different patients has been tested by the two methods (14). These samples were selected to cover the entire working range of the method, that is, all three blood types, type A, B, and AB, and should represent the distribution of blood types expected in routine application of the method. However, more blood samples obtained from type AB or B cats were included in the study to assist recognition of any potential typing problems.

Sensitivity (Se), specificity (Sp), positive likelihood ratio (PLR), negative likelihood ratio (NLR), positive predictive value (PPV), negative predictive value (NPV), and overall accuracy for GEL were calculated, with TUBE results considered the gold standard. For this method's comparison study, samples were preferentially obtained from type AB or B cats to assist in recognizing any unique potential typing problem. Therefore, as the sample sizes for each blood type used in this study did not reflect the real prevalence of AB blood types in the tested feline population, to evaluate use as a diagnostic test, the real data prevalence from a previous study performed on 140 cats living in the same area and tested by the same authors of this study was used (5), that is, a prevalence of 90.7% for type A, 7.1% for type B, and 2.1% for type AB.

An interrater agreement, unweighted κ statistic (κ) was calculated with 95% confidence interval (95% CI) to evaluate agreement between the two testing methods in detecting blood type (15). The level of agreement based on value of κ was scored according to the following guidelines (16):

- <0.20: Poor
- 0.21–0.40: Fair
- 0.41–0.60: Moderate
- 0.61–0.80: Good
- 0.81–1.00: Very good

Relationship between results of FIV-FeLV test and samples showing mixed-field pattern in GEL technique was tested with Fisher exact test with significance set at *P* < 0.05.

All statistical analyses were performed using a statistical software package (MedCalc Software, version 19.1.3; Mariakerke, Belgium).

To test the intra-assay precision, the GEL was performed six times on the same day on a single sample of type A, B, and AB. All tests were run in the same laboratory and interpreted by the same technician blinded to previous results.

Five samples of each of type A, B, and AB were tested in a blinded fashion by three different operators (a technician, a veterinarian, and a student) to establish the interassay accuracy of GEL test results.

One sample of each blood type stored at room temperature and at 4 ± 2°C was analyzed with GEL at T0, after 24 and 48 h, and after 1, 2, 3, and 4 weeks of storage.

At D0 (the day of collection of the unit) and on D28 (day of the expiration of the unit), GEL was performed on five samples of type A citrate phosphate dextrose adenine (CPDA) anticoagulated blood drawn from both fresh and stored whole-blood units.

## Results

One hundred forty-three blood samples were tested in this study and were from 127 European domestic shorthair, 7 Ragdolls, 3 Persians, 2 Maine Coons, 1 Norwegian forest cat, 1 British shorthair cat, 1 Sphynx, and 1 Siamese. Fifty-seven samples were from female cats and 86 from male, aged between 1 month and 18 years [mean, 4.3 (SD 4.1) years]. Ten cats were blood recipients, 13 were blood donors, 15 were blood typed for breeding purposes, 18 as a preoperative check, and 87 cats were blood typed as part of an epidemiological study (data/results not shown).

Mean PCV was 30.3% (SD, 9.9%; min–max, 9.0–46.7%); three samples showed autoagglutination, and 15 samples were from anemic cats [PCV <24%; mean, 17.2% (SD, 5.4%); min–max, 9.0–23.9%].

Of 143 samples typed with TUBE, 98 (68.5%) were type A, 25 (17.5%,) type B, and 20 (14.0%) type AB. Most agglutination reactions were strongly positive (4+ or 3+). All types B and AB samples were confirmed by results of backtyping, with strong agglutination reaction and no agglutination in types B and AB plasma samples, respectively, crossmatched against type A RBCs.

With GEL, 115/143 samples (80.4%) gave concordant results with TUBE; a mixed-field agglutination pattern (presence of a layer of RBCs at the top and bottom of A or B gel column, Figure 4) was seen in 27 samples (18.9%), and one type B sample was misidentified as type AB (Table 1). As in TUBE, most agglutination reactions were strongly positive (4+ or 3+). Results of Se, Sp, PLR, NLR, PPV, NPV, and accuracy are reported in Table 2. Strength of agreement of two methods in blood typing when mixed-field pattern results were considered positive was good (κ= 0.67; standard error, 0.05; 95% CI, 0.56–0.77).

**Table 1:** Feline blood type results of 143 EDTA blood samples tested with agglutination on TUBE (gold standard) and GEL techniques where gel mixed-field pattern results were considered positive results.

**Gel agglutination**	**Tube agglutination (gold standard)**			
	**A**	**B**	**AB**	**Total (%)**
A	76	0	0	76 (53.1)
B	0	20	0	20 (14.0)
AB	22^*^	5^**^	20***	47 (32.9)
Total (%)	98 (68.5)	25 (17.5)	20 (14.0)	143 (100)

* *In all these samples, a mixed-field pattern in B GEL column was identified*.

** In four of these samples and in ***one of these samples, a mixed-field pattern in A GEL column was identified and interpreted as a positive result, that is, test positive for agglutination.

**Table 2:** Diagnostic performances of a GEL technique compared to a TUBE technique in feline AB system blood typing of 143 feline whole-blood samples anticoagulated with EDTA, considering GEL mixed-field pattern results as positive results.

**Statistics**	**Blood type with GEL (considering mixed-field pattern as positive results)**					
	**compared to TUBE technique**					
	**A**		**B**		**AB**	
	**Value (%)**	**95% CI**	**Value (%)**	**95% CI**	**Value (%)**	**96% CI**
Sensitivity (Se)	77.55	68.01–85.36	80.00	59.30–93.17	100.00	83.16–100.00
Specificity (Sp)	100.00	92.13–100.00	100.00	96.92–100.00	78.05	69.69–85.01
Positive likelihood ratio (PLR)	–	–	–	–	4.56	3.26–6.36
Negative likelihood ratio (NLR)	0.22	0.16–0.32	0.20	0.09–0.44	0.00	–
Positive predictive value (PPV)	100.00	–	100.00	–	9.90	6.54–12.00
Negative predictive	31.35	24.02–39.76	98.49	96.76–99.31	100.00	–
Accuracy	79.64	72.10–85.91	98.58	95.01–99.82	78.51	70.87–84.93

Twenty of 27 samples with GEL mixed-field pattern were tested for FIV and FeLV infection, and four samples tested FIV seropositive. Thirty-six of 116 samples with normal GEL pattern were tested; one was FIV seropositive, and one FeLV seropositive. Relationship between retrovirus seropositivity and presence of GEL mixed-field pattern was not significant (*P*= 0.17).

If GEL mixed-field pattern results were interpreted as negative results (as gold standard method showed no agglutination), 141/143 (98.6%) samples showed concordant results (Table 3) with Se, Sp, PLR, NLR, PPV, NPV, and accuracy as reported in Table 4. One TUBE type B sample was identified as type AB by GEL and one TUBE type AB as B by GEL. Strength of agreement of two methods in blood typing was very good (κ = 0.97; standard error, 0.02; 95% CI:0.93–1.00).

**Table 3:** Feline blood type results of 143 EDTA blood samples with agglutination on tube (gold standard) and with gel techniques where GEL mixed-field pattern results considered negative results.

**Gel agglutination**	**Tube agglutination (gold standard)**			
	**A**	**B**	**AB**	**Total (%)**
A	98^*^	0	0	98 (68.5)
B	0	24^**^	1^***^	25 (17.5)
AB	0	1	19	20 (14.0)
Total	98 (68.5)	25 (17.5)	20 (14.0)	143 (100)

* *Twenty-one samples showed a mixed pattern in the B GEL column and were interpreted as negative results/absence of agglutination*.

** *Four of these samples showed a mixed pattern in the A GEL column and were interpreted as negative results, that is, absence of agglutination*,

*** *This sample showed a mixed pattern in the A GEL column and was interpreted as negative*.

**Table 4:** Diagnostic performances of a GEL technique compared to a TUBE technique in feline AB system blood typing of 143 feline whole-blood samples anticoagulated with EDTA, where GEL mixed pattern results were considered as negative results.

**Statistics**	**Blood type with GEL (considering mixed-field pattern as negative**					
	**results) compared to TUBE technique**					
	**A**		**B**		**AB**	
	**Value**	**95% CI**	**Value**	**95% CI**	**Value**	**96% CI**
	**(%)**		**(%)**		**(%)**	
Sensitivity (Se)	100.00	96.31–100.00	96.00	79.65–99.90	95.00	75.13–99.87
Specificity (Sp)	100.00	92.13–100.00	99.15	95.37–99.98	99.19	95.55–99.98
Positive likelihood ratio (PLR)	–	–	113.28	16.06–798.86	116.85	16.55–825.09
Negative likelihood ratio (NLR)	0.00	–	0.04	0.01–0.28	0.05	0.01–0.34
Positive predictive value(PPV)	100.00	–	89.65	55.11–98.39	71.48	26.20–94.65
Negative predictive value (NPV)	100.00	–	99.69	97.94–99.95	99.89	99.27–99.98
Accuracy	100.00	97.45–100.00	98.93	95.55–99.92	99.10	95.83–99.95

All anemic and autoagglutinated samples were correctly typed by GEL.

Single samples of types A, B, and AB blood typed multiple times with GEL were always correctly blood typed. The GEL method showed 100% intra-assay accuracy.

All five samples of types A, B, and AB were correctly identified giving an excellent interassay accuracy. Interassay accuracy was not affected by using three different operators.

Blood samples stored at room temperature and at 4 ± 2°C for up to 1 month were correctly typed by GEL—as were all samples from whole-blood units anticoagulated with CPDA1 on D0 and on D28 of storage.

## Discussion

Feline transfusion medicine has grown rapidly in the last decades, and today, many sick cats receive blood transfusion, and many healthy cats donate blood. Even a small quantity of incompatible blood in the first blood transfusion could cause an acute hemolytic transfusion reaction that could be fatal for type B cats receiving type A blood (17). In all other incompatible blood transfusions, the failure to type blood correctly could result in a less effective transfusion, wasting a very precious and limited resource (18). Therefore, it is imperative to have sensitive and specific blood-typing methods in both emergency situations, in which fast and precise immunomigration tests are needed, and in routine and epidemiological settings, where high sensitivity and specificity can be combined with easy, rapid and economic test characteristics.

Accurate blood typing is often difficult to achieve in sick patients. Many feline patients received Whole Blood (WB) transfusion for anemia. Anemia may be severe in these cases, and sometimes these patients show persistent autoagglutination of blood samples. Severe anemia and autoagglutination can pose problems in blood typing, especially when using agglutination on cards or tests based on RBCs migration (7, 9, 19). The TUBE, technique overcomes problems related to severe anemia, as centrifugation concentrates RBCs in the sample, and relatively few RBCs are needed to run these tests. All anemic samples in our study were correctly typed, even when PCV was as low as 9%. Autoagglutination can be detected in the TUBE and GEL methods if a control reaction with saline or PBS is run to reveal false-positive results due to autoagglutinated samples. In addition, the TUBE method is useful in autoagglutination as the few non-agglutinating RBCs in these samples are sufficient to detect the blood type, as confirmed by our study when three autoagglutinated samples were correctly typed by GEL.

The most important problem with GEL in AB feline group blood typing highlighted by this study was the occasionally mixed-field pattern result, which makes interpretation difficult without a reference to another method of typing. Mixed-field pattern results have been reported by other researchers in a limited number of veterinary publications. In a sample from a cat with FeLV-related anemia, the GEL test reaction showed a split population of RBCs in the anti-B column, making the results inconclusive (9). Our results suggest that retrovirus infections, that is, FIV or FeLV infections, do not result in GEL mixed-field patterns.

In a few cases, Euler et al. (12) observed an unexplained split reaction in Kai canine blood typing, with the majority of cells located on top of the gel despite a few RBCs being pelleted. These cases were classified as 4+ results. These dogs had not been previously transfused, nor had any illness (12). In human medicine, GEL mixed-field patterns arise from (I) *mixed RBCs populations* from recent blood transfusions (presence of circulating donor red cells in recipient), transplanted bone marrow or peripheral blood stem cells of a different ABO type, exchange transfusions, fetal–maternal hemorrhage, blood group chimerism in fraternal twins, mosaicism arising from dispermy (20, 21); (II) false-positive reactions when *incompletely clotted serum* is used (21); (III) *various diseases that alter RBC antigens* and result in progressively weaker reactions or additional acquired pseudoantigens (e.g., leukemia, Hodgkin disease, thalassemia) (22); (IV) some *ABO subgroups* (21). Considering that 18.9% of the samples analyzed in this study with GEL showed a mixed-field pattern, it is improbable that all these patients were affected by chimerism or mosaicism or had received recent blood transfusion that transiently changed their blood type. As the type B plasma used as anti–type A reagent was the same in both methods (GEL and TUBE) and for all samples, this should not be the cause of mixed-field pattern. As health information related to blood samples analyzed in this study was collected retrospectively, we have limited information on some patients, and apart from the possible presence of diseases such as FeLV or FIV infection, we have limited data on presence of neoplasia, feline immune-mediated hemolytic anemia, or severe inflammation, which could alter RBC antigens (9, 23). Therefore, the possibility that some disease could be the cause of this mixed-field pattern cannot be excluded. It is also possible that subtle or microscopic autoagglutination that was not detected by TUBE resulted in the GEL mixed-field pattern due to a reaction with RBC antigens from either a blood type outside the AB blood type system such as Mik (24) or, as previously demonstrated, the presence of natural occurring alloantibodies outside the AB blood group system (25, 26). To prevent nonspecific agglutination reactions causing blood type misclassification, monoclonal antibodies to type A RBC would be the ideal reagent for the future use in GEL methods.

The characteristic of the samples could be responsible for the mixed-field pattern. Mixed-field reactions can occur when incompletely clotted serum is used in the gel test in human medicine. Fibrin strands in serum may trap unagglutinated RBCs, forming a thin line at the top of the gel, whereas other unagglutinated cells pass through the gel during centrifugation and fall to the bottom of the microtube (21). Rouleaux caused by serum or plasma with abnormally high concentrations of protein may produce hazy reactions or false-positive results (21). Presence of rouleaux is a frequent hematological feature in diseased (but also in many healthy) cats (27). Future studies should therefore explore reasons for GEL mixed-field patterns looking for the presence of hyperproteinemia or presence of rouleaux and trying to repeat the test in samples with GEL mixed-field pattern after washing of RBCs to disperse rouleaux as performed in the TUBE technique.

However, if the mixed-field patterns were considered as negative results, there were only two discordant results between TUBE and GEL. One was a TUBE type AB cat typed as B by GEL. This sample come from a young female cat with recent Giardia infestation that was blood typed before surgery for spaying and was FIV and FeLV negative. The second discordant result was a type B sample misclassified as type AB by GEL from a healthy young cat blood typed before routine spaying. Unfortunately, we have no additional information on this cat, and it was not possible to test for FIV and FeLV infections as there was insufficient sample. Misclassifying a type B cat as type AB could have serious clinical consequences if testing a blood recipient, as a type AB cat could receive type A blood with no (or limited) clinical sequela, but in a type B cat, a small amount of type A blood could result in a fatal hemolytic reaction. However, following good practices, crossmatching should be done before every blood transfusion, which would reveal a blood incompatibility between types A and B blood, and other blood types such as Mik (24), to avoid hemolytic transfusion reactions.

Although the TUBE method is considered the gold standard technique in feline blood typing (8, 9), the GEL test provides major advantages in AB blood typing compared with traditional tube technology (21, 28). The most important advantages in veterinary medicine appear to be (I) *rapidity* as, with GEL, the blood type was established in half the time of the TUBE method (mostly because GEL does not require the washing of RBCs, which reduced the overall time to obtain blood type): this advantage is particularly important in emergency situation or in epidemiological studies when many samples are analyzed as a batch; (II) *stable, well-defined endpoints* of the agglutination reaction that can be reviewed for up to 3 days: the result can be photographed and archived, viewed by multiple technicians at different times, whereas, with TUBE, the endpoint reaction is stable for only a few hours; (III) more *objective and consistent results* as the endpoint agglutination results are more clear and simple to read, grade, and interpret; (IV) *standardization*, because there is no tube shaking to resuspend the RBC button, the variability associated with the physical resuspension of RBC buttons after centrifugation and the subsequent interpretation of hemagglutination reactions are reduced, permitting more reproducible test results; (V) *reduced volume of sample and reagents* as only 10 μL of the RBC pellet is required: this is particularly useful when working with small animals such as cats for which the blood sample volume is often limited. In addition, type B plasma sample used as anti-A reagent can be saved and used at a quarter of the volume used in TUBE, preserving an important biological reagent; (VI) *fewer operator interventions*, thus reducing technical error. For all these reasons, some veterinary laboratories, particularly those working with large number of samples or needing rapid results for emergency situations, might prefer GEL over TUBE in blood typing feline samples.

This study did not evaluate the agreement between methods for agglutination strength reactions. In tests based on agglutination using *T. vulgaris* lectin as anti-B reagent and type B plasma as anti-A reagent, the reactions are usually strong, 3+ or 4+, as seen in the samples analyzed in this study. As the objective of the study was to compare GEL to TUBE technique, most samples had incomplete clinical information. Therefore, we cannot rule out the possibility that some animals may have had infections or diseases other than retrovirus infections, such as neoplasia, feline immune-mediated hemolytic anemia, or severe inflammation, which could alter RBC antigens.

In conclusion, if the same anti-A and anti-B reagents are used as in TUBE testing, the GEL column technique is a sensitive and specific method for typing feline blood samples, with a number of advantages over the TUBE technique. Until additional studies improve our understanding of the significance of mixed-field pattern in GEL technology, a mixed-field pattern should be considered as a negative result in veterinary transfusion medicine. As TUBE is the only method that has 100% sensitivity and specificity in detecting B and AB blood types, crossmatching before blood transfusion remains key to prevent fatal incompatible transfusion reactions.

## Data Availability Statement

The datasets generated for this study are available on request to the corresponding author.

## Ethics Statement

Ethical review and approval for this animal study was not required according to our institutional legislation because only leftover blood samples sent for routine diagnostic work were used. Written informed consent for further use of surplus material was obtained from the owner of the animals for the samples used in this study.

## Author's Note

This research was presented in part as poster at 60th American Association Veterinary Laboratory Diagnosticians (AAVLD)/121st United States Animal Health Association (USAHA) Annual Meeting, San Diego, CA, USA, 12–18 October 2017.

## Author Contributions

ES, RP, and DP contributed conception and design of the study; LB performed the laboratory analysis and organized the database; ES performed the statistical analysis, wrote the first draft of the manuscript. All authors contributed to manuscript revision, read, and approved the submission version.

## Conflict of Interest

The authors declare that the research was conducted in the absence of any commercial or financial relationships that could be construed as a potential conflict of interest.
